# Supplementary material for: Impact of Sociodemographic, Premorbid, and Injury-Related Factors on Patient-Reported Outcome Trajectories after Traumatic Brain Injury (TBI)
Source: J Clin Med. 2023 Mar 14;12(6):2246. doi: 10.3390/jcm12062246 (PMC10052290; doi:10.3390/jcm12062246)
Supplement: Supplementary file 1 [file jcm-12-02246-s001.zip › S2_Tables_S1_S2.pdf]

Table S1. Prediction of symptom trajectories (PHQ-9, GAD-7, PCL-5, RPQ) for the 4-class solution of model 1 (random intercept, fixed slope)

		Ref. = Stable good health					
		Persistent health impairments		Deteriorating health		Improving health	
		OR [95% CI]	*p*	OR [95% CI]	*p*	OR [95% CI]	*p*
Age (in years)		**0.96 [0.94, 0.98]**	**<0.001**	0.99 [0.97, 1.01]	0.258	0.99 [0.97, 1.00]	0.150
Sex (ref. = Female)	Male	0.84 [0.57, 1.23]	0.366	0.76 [0.53, 1.09]	0.129	0.83 [0.61, 1.13]	0.233
Education level (ref. = College/university)	None/primary school	**2.65 [1.54, 4.57]**	**<0.001**	1.26 [0.70, 2.27]	0.432	1.57 [0.97, 2.53]	0.065
	At least secondary/high school	1.49 [0.96, 2.31]	0.079	1.11 [0.72, 1.70]	0.644	1.10 [0.74, 1.63]	0.632
	Post-high school training	1.25 [0.74, 2.11]	0.405	1.42 [0.89, 2.28]	0.142	1.31 [0.85, 2.03]	0.225
Employment status (ref. = Full-time employed)	Part-time employed	0.76 [0.43, 1.35]	0.353	**1.81 [1.10, 3.00]**	**0.020**	0.95 [0.58, 1.58]	0.856
	In training	0.56 [0.28, 1.14]	0.111	1.26 [0.62, 2.57]	0.517	0.91 [0.49, 1.67]	0.750
	Unemployed	1.69 [0.98, 2.90]	0.059	1.52 [0.85, 2.74]	0.158	1.55 [0.95, 2.53]	0.078
	Retired	1.16 [0.58, 2.30]	0.678	0.71 [0.36, 1.40]	0.318	0.93 [0.53, 1.63]	0.795
Marital status (ref. = Married)	Never been married	0.69 [0.40, 1.19]	0.182	0.66 [0.33, 1.30]	0.226	0.90 [0.51, 1.58]	0.718
	Living together/common law	1.04 [0.56, 1.91]	0.906	0.91 [0.49, 1.66]	0.748	0.80 [0.45, 1.43]	0.449
	Divorced/separated	1.64 [0.89, 3.02]	0.115	1.30 [0.69, 2.45]	0.419	1.35 [0.78, 2.33]	0.277
	Widowed	0.59 [0.15, 2.38]	0.460	1.23 [0.44, 3.40]	0.694	1.06 [0.48, 2.33]	0.880
Living alone (ref. = No)	Yes	1.04 [0.62, 1.75]	0.867	0.82 [0.47, 1.43]	0.484	0.97 [0.61, 1.55]	0.904
Physical health Status (ASA) (ref. = Normal healthy)	Mild disease	1.09 [0.70, 1.71]	0.688	1.14 [0.77, 1.71]	0.511	1.26 [0.87, 1.84]	0.227
	Severe disease	1.34 [0.68, 2.65]	0.402	1.50 [0.71, 3.15]	0.287	1.25 [0.70, 2.24]	0.454
Psychol. problems (ref. = No)	Yes	**3.43 [2.18, 5.41]**	**<0.001**	**2.00 [1.20, 3.31]**	**0.007**	**2.20 [1.46, 3.30]**	**<0.001**
TBI history (ref. = No)	Yes	0.97 [0.54, 1.71]	0.904	1.15 [0.64, 2.06]	0.634	1.13 [0.71, 1.78]	0.610
Cause of injury (ref. = Road traffic incident)	Incidental fall	0.77 [0.51, 1.16]	0.206	0.73 [0.50, 1.06]	0.098	0.97 [0.69, 1.37]	0.857
	Other	0.99 [0.60, 1.64]	0.972	0.95 [0.57, 1.59]	0.837	0.79 [0.50, 1.26]	0.327
Clinical care pathways (ref. = ER)	Admission	0.84 [0.47, 1.50]	0.556	1.52 [0.76, 3.04]	0.241	0.61 [0.37, 1.01]	0.052
	ICU	0.54 [0.23, 1.25]	0.150	**2.45 [1.07, 5.62]**	**0.035**	**0.48 [0.24, 0.94]**	**0.034**
Length of hospital stay (days)		**0.99 [0.97, 1.00]**	**0.033**	1.00 [1.00, 1.01]	0.294	1.00 [0.99, 1.01]	0.392
Injury Severity Score (ISS)		0.99 [0.97, 1.01]	0.424	0.99 [0.97, 1.01]	0.172	1.01 [0.99, 1.02]	0.229

Brain Injury Score (AIS)		0.88 [0.68, 1.15]	0.352	1.07 [0.86, 1.33]	0.560	1.08 [0.87, 1.33]	0.484
TBI Severity Classification (ref. = Uncomplicated mild)	Complicated mild	1.56 [0.94, 2.59]	0.086	1.19 [0.72, 1.97]	0.505	0.84 [0.56, 1.26]	0.386
	Moderate	1.37 [0.61, 3.08]	0.442	0.91 [0.43, 1.92]	0.810	0.75 [0.37, 1.50]	0.414
	Severe	1.17 [0.54, 2.55]	0.690	1.08 [0.52, 2.25]	0.827	0.78 [0.41, 1.46]	0.431
GOSE (3 months) (ref. = 7/8 (Lower/ Upper GR))	2/3 (VS/Lower SD)	**17.88 [8.45, 37.85]**	**<0.001**	**3.15 [1.55, 6.41]**	**0.002**	**7.29 [4.00, 13.31]**	**<0.001**
	4 (Upper SD)	**18.42 [9.84, 34.47]**	**<0.001**	**3.88 [2.24, 6.69]**	**<0.001**	**5.62 [3.25, 9.71]**	**<0.001**
	5 (Lower MD)	**11.12 [5.92, 20.89]**	**<0.001**	**3.92 [2.27, 6.77]**	**<0.001**	**5.74 [3.22, 10.22]**	**<0.001**
	6 (Upper MD)	**5.10 [2.93, 8.88]**	**<0.001**	**2.88 [1.77, 4.68]**	**<0.001**	**5.07 [3.39, 7.58]**	**<0.001**

Note. SD = Severe disability, MD = moderate disability, GR = good recovery, OR = Odds Ratio, CI = confidence interval, p = p-value. Values in **bold** are significant at $p < 0.05$.

Table S2. Prediction of quality of life trajectories (QOLIBRI-OS, SF-12 MCS, SF-12 PCS) for the 4-class solution of model 1 (random intercept, fixed slope).

		Ref. = Stable good health					
		Persistent health impairments		Deteriorating health		Improving health	
		OR [95% CI]	*p*	OR [95% CI]	*p*	OR [95% CI]	*p*
Age (in years)		1.00 [0.98, 1.01]	0.465	1.00 [0.97, 1.03]	0.932	1.00 [0.98, 1.03]	0.759
Sex (ref. = Female)	Male	**0.61 [0.48, 0.79]**	**<0.001**	1.08 [0.63, 1.85]	0.779	**0.50 [0.29, 0.85]**	**0.010**
Education level (ref. = College/university)	None/primary school	**2.26 [1.53, 3.36]**	**<0.001**	1.79 [0.82, 3.91]	0.145	1.36 [0.66, 2.79]	0.407
	At least secondary/high school	**1.53 [1.11, 2.11]**	**0.010**	1.38 [0.74, 2.57)	0.312	0.73 [0.38, 1.38]	0.331
	Post-high school training	**1.56 [1.11, 2.20]**	**0.011**	1.23 [0.60, 2.49]	0.570	1.27 [0.65, 2.49]	0.482
Employment status (ref. = Full-time employed)	Part-time employed	0.95 [0.66, 1.39]	0.805	1.21 [0.55, 2.63]	0.637	1.28 [0.58, 2.82]	0.542
	In training	0.56 [0.30, 1.04]	0.066	0.27 [0.01, 6.30]	0.417	1.69 [0.57, 4.99]	0.339
	Unemployed	**1.87 [1.25, 2.80]**	**0.002**	1.69 [0.74, 3.86]	0.216	1.41 [0.56, 3.51]	0.465
	Retired	0.68 [0.44, 1.07]	0.098	0.71 [0.30, 1.69]	0.443	0.94 [0.38, 2.36]	0.900
Marital status (ref. = Married)	Never been married	0.73 [0.48, 1.10]	0.136	1.00 [0.42, 2.39]	0.997	0.71 [0.30, 1.72]	0.452
	Living together/common law	1.07 [0.69, 1.66]	0.752	0.67 [0.27, 1.67]	0.384	0.64 [0.14, 2.93]	0.566
	Divorced/separated	1.14 [0.72, 1.78]	0.579	0.54 [0.11, 2.65]	0.446	1.11 [0.47, 2.62]	0.820
	Widowed	1.48 (0.81, 2.69]	0.198	2.49 [0.85, 7.29]	0.095	0.41 [0.01, 15.86]	0.633
Living alone (ref. = No)	Yes	**1.60 [1.11, 2.30]**	**0.011**	0.99 [0.47, 2.06]	0.978	1.48 [0.72, 3.03]	0.287
Physical health Status (ASA) (ref. = Normal healthy)	Mild disease	1.17 [0.86, 1.59]	0.307	1.17 [0.65, 2.14]	0.598	1.27 [0.71, 2.27]	0.416
	Severe disease	**2.22 [1.36, 3.64]**	**0.001**	1.49 [0.56, 3.97]	0.427	1.45 [0.56, 3.74]	0.445
Psychol. problems (ref. = No)	Yes	**2.25 [1.59, 3.19]**	**<0.001**	1.41 [0.70, 2.85]	0.335	**2.57 [1.37, 4.83]**	**0.003**
TBI history (ref. = No)	Yes	0.96 [0.63, 1.47]	0.864	1.62 [0.79, 3.33]	0.187	1.46 [0.66, 3.26]	0.351
Cause of injury (ref. = Road traffic incident)	Incidental fall	0.82 [0.62, 1.10]	0.190	0.60 [0.36, 1.02]	0.059	0.97 [0.55, 1.71]	0.912
	Other	0.88 [0.60, 1.30]	0.525	0.52 [0.24, 1.12]	0.094	1.07 [0.44, 2.56]	0.887
Clinical care pathways (ref. = ER)	Admission	0.71 [0.47, 1.09]	0.116	3.62 [0.79, 16.61]	0.098	2.12 [0.27, 16.97]	0.477
	ICU	0.63 [0.35, 1.15]	0.134	4.41 [0.80, 24.30]	0.088	2.51 [0.26, 24.62]	0.428
Length of hospital stay (days)		1.00 [0.99, 1.01]	0.741	1.00 [0.99, 1.02]	0.478	**0.98 [0.97, 1.00]**	**0.048**
Injury Severity Score (ISS)		1.00 [0.99, 1.02]	0.762	0.98 [0.96, 1.01]	0.212	1.01 [0.99, 1.03]	0.346

Brain Injury Score (AIS)		0.95 [0.80, 1.13]	0.549	0.94 [0.68, 1.32]	0.738	1.19 [0.84, 1.69]	0.324
TBI Severity Classification (ref. = Uncomplicated mild)	Complicated mild	0.93 [0.65, 1.32]	0.666	1.19 [0.60, 2.37]	0.612	0.61 [0.28, 1.33]	0.212
	Moderate	0.86 [0.50, 1.49]	0.598	1.22 [0.31, 4.80]	0.778	0.90 [0.30, 2.67]	0.845
	Severe	0.79 [0.47, 1.31]	0.358	1.52 [0.52, 4.47]	0.443	0.76 [0.28, 2.04]	0.579
GOSE (3 months) (ref. = 7/8 (Lower/ Upper GR))	2/3 (VS/Lower SD)	**27.24 [15.71, 47.22]**	**<0.001**	**3.92 [1.63, 9.43]**	**0.002**	**24.32 [9.93, 59.54]**	**<0.001**
	4 (Upper SD)	**13.47 [8.70, 20.84]**	**<0.001**	1.97 [0.91, 4.27]	0.086	**5.94 [2.25, 15.74]**	**<0.001**
	5 (Lower MD)	**7.24 [4.60, 11.41]**	**<0.001**	1.60 [0.65, 3.96]	0.305	**5.93 [2.22, 15.83]**	**<0.001**
	6 (Upper MD)	**4.23 [2.90, 6.15]**	**<0.001**	1.61 [0.83, 3.09]	0.157	**5.60 [2.70, 11.59]**	**<0.001**

Note. SD = Severe disability, MD = moderate disability, GR = good recovery, OR = Odds Ratio, CI = confidence interval, p = p-value. Values in **bold** are significant at $p < 0.05$.
